# Nanoporous Anodic Aluminum-Iron Oxide with a Tunable Band Gap Formed on the FeAl_3_ Intermetallic Phase

**DOI:** 10.3390/ma13163471

**Published:** 2020-08-06

**Authors:** Paulina Chilimoniuk, Robert P. Socha, Tomasz Czujko

**Affiliations:** 1 Institute of Materials Science and Engineering, Faculty of Advanced Technology and Chemistry, Military University of Technology, Kaliskiego 2 Street, 00-908 Warszawa, Poland; 2 Jerzy Haber Institute of Catalysis and Surface Chemistry, Polish Academy of Sciences, Niezapominajek 8, 30-239 Krakow, Poland; ncsocha@cyf-kr.edu.pl

**Keywords:** anodic oxides, self-organization, photocatalysis, band gap, anodization

## Abstract

Nanostructured anodic oxide layers on an FeAl_3_ intermetallic alloy was prepared by two-step anodization in 20 wt.% H_2_SO_4_ at 0 °C. The obtained anodic oxide coating was subjected to phase and chemical composition analysis using XPS and XRD techniques. An analysis of the band gap of individual coatings was also performed. The applied parameters of the anodization process were determined, enabling the formation of a nanostructured coating on the FeAl_3_ intermetallic alloy. Tests were carried out on samples produced at a voltage between 10 V and 22.5 V in 2.5 V steps. The produced coatings were subjected to an annealing process at 900 °C for 2 h in an argon protective atmosphere. Moreover, the influence of the substrate chemical composition on the chemical and phase composition of the anodic oxide are discussed. Band gaps of 2.37 eV at 22.5 V and 2.64 eV at 10 V were obtained directly after the anodizing process. After applying the heat treatment, band gap values of 2.10 eV at 22.5 Vand 2.48 eV for the coating produced at 10 V were obtained.

## 1. Introduction

The electrochemical anodization process is one of several processes that produces nanometric coatings of metal oxides on the surface of various types of materials.The process of electrochemical anodization is commonly understood to be a process that uses electrochemical phenomena that involve producing an oxide nanolayer under the influence of an electric field on the surface of the metal placed in the electrolyte. This process is carried out in simple two-electrode systems, where the anode is the base material for the growth of the nanometric anodized layer.This process can be carried out both on pure metals and their alloys, and is of particular relevance for construction materials, for example metals such as CuAl [1], V [2], and intermetallic-based alloys, such as Ni_3_Al [3,4], TiAl [5,6,7]. In the past, this technology was mainly used to protect the material against corrosion as well as for decorative purposes. Despite this, over the years, other application possibilities for theoxides obtained in the anodization process have been noticed. An example is anodic aluminum oxide, which is the basic material for the production of matrices used in the design of various types of nanostructures, such as nanotubes, nanocaps, and nanowires. Additionally, anodic oxides reused in implantology to create highly developed surfaces for improved tissue adhesion and growth. On the other hand, when anodic oxides are prepared on various types of machine components, they prevent premature wear and destruction [8,9,10,11,12]. This method allows designing a variety of nanostructured materials with different oxide morphologies by selecting the appropriate process parameters. It should be noted that each attempt to carry out this process requires individual optimization of parameters for each type of substrate material. The parameters that determine the morphology of the anodic oxide produced include the type of aqueous or non-aqueous electrolyte solutions and their concentration and temperature as well as the potential and duration of the electrochemical anodization process [13]. Parameters that describe the geometry of nanostructured anodic oxides, include the average pore diameter, average distance between pore centers, thickness of the oxide coating, and thickness of the barrier layer, which separates the bottom of the resulting pores from the substrate material; this is especially true for anodic aluminum oxide, which is a typical material produced by anodization [14,15,16]. Nanolayers owe their great interest to their specific functional properties, classifying them to the group of engineering nanomaterials, which is widely used in many branches of technology and industry. As is known, some materials tend to spontaneously form a protective oxide film, the so-called self-passivation. Examples of such materials are aluminum, nickel, or titanium. Because of its very good passivation ability, aluminum, which is largely present in the alloys based on the intermetallic phase of the Fe-Al system, largely contributes to their heat resistance. However, not all materials have this feature. Therefore, as the protection of metals and their alloys against corrosion in industrial applications, artificial passivation is used, obtaining an oxide layer with appropriate properties, often better than natural passive layers [17]. For this purpose, conversion coatings or an anodic oxidation process are commonly used.

Therefore, in addition to aluminum, the electrochemical oxidation process was also performed on an alloy based on an FeAl intermetallic alloy. This process was carried out for 60 s using 20% aqueous sulfuric acid solution as an electrolyte. The nanoporous oxide structure was obtained at 0 °C. This structure was characterized by the variability of geometrical parameters depending on the applied voltage in the range from 5 V to 20 V with a step size of 2.5 V [15]. As the research shows, the obtained anodic oxide has an amorphous structure, and after heat treatment, it transforms into the crystal lattice of the FeAl_2_O_4_ spinel phase. In addition, a decrease in the band gap of the produced oxide structure was noted along with an increase in the anodization potential. The oxide coating produced at a voltage of 5 V has a bandgap of approximately 5.0 eV, while the band gap for the coating manufactured at 17.5 V is 2.09 eV [15,18]. This can prove the possibility of controlling the value of the band gap through the selection of technical process parameters. This feature is extremely interesting from the point of view of photocatalytic water splitting, which prompts further research of this intermetallic phase. In addition, an increase in the morphological parameters of nanopores in the oxide structure obtained on the FeAl intermetallic alloy was observed with an increase in the applied voltage [15]. Similar effects were observed for the structures produced by anodizing aluminum at various temperatures using 20% H_2_SO_4_ as well as 0.3 M oxalic acid, where in both cases, an increase in pore diameter and distance between the pores was observed along with an increase in the potential [18,19].

It was also observed that at elevated voltages, the pore diameters were much larger in anodic oxides formed on an FeAl intermetallic alloy than in the case of anode oxides formed on high-purity aluminum in the same voltage range in sulfuric acid [20] and oxalic acid [19]. In addition, the distance between the pores was larger than that observed for high-purity aluminum at the same voltage in the case of anodizing carried out in sulfuric [20] and oxalic acids [21]. The distance between the pores is closely related to the density of the pores. The resulting larger distance between the pores in the layer of anodic oxide formed on FeAl intermetallic alloys than that on pure Al is related to the higher density of pores in the latter case.

Unfortunately, it must be noted that the anodic oxide produced on FeAl intermetallic alloys is unstable and delaminates during the photocatalytic process of water splitting. An appropriate ratio of aluminum to iron concentrations in the alloy should ensure a satisfactory degree of ordering of porous structures, a high durability and the required band gap. All these indicated material properties of oxide layers are determined by the composition of the oxide mixture formed in the anodization process. In turn, this is not only the result of the structure and composition of the base material but also the result of the parameters of the anodization process. Considering that alumina produced on pure Al has a satisfactory durability but a high band gap, it seems reasonable to analyze the ability to produce anodic oxide coatings on a substrate based on intermetallic alloys from the Fe-Al system with a reduced iron concentration [18]. An example of such a material is an alloy based on the FeAl_3_ intermetallic.

Literature studies indicate an almost complete lack of work dedicated to FeAl_3_ anodization. Therefore, the need to thoroughly investigate the possibility of producing a nanoporous anode layer on the FeAl_3_ intermetallic alloy substrate as a potential new photocatalytic material is justified [18]. Recently, we published a paper on the structure of anodic oxides produced on an FeAl_3_ substrate. In the current paper, we present and discuss the influence of applied process parameters as well as substrate chemical composition on the phase and chemical composition of the coatings directly after anodization and heat treatment. An analysis of the band gap of individual coatings resulting from the obtained structure is presented. Moreover, the ability to control the band gap value of the produced anodic oxide coating is discussed.

## 2. Materials and Methods

The FeAl_3_ intermetallic alloy was cut into coupons with a thickness of 0.9 mm. The chemical composition of the FeAl_3_ intermetallic alloy was 22.47 at.% Fe and 77.53 at.% Al.The chemical composition of substrate material was measured using EDAX type energy dispersion microanalysis device attached to the field emission scanning electron microscopy (FE-SEM, Quanta 3D FE-SEM, FEI, Hillsboro, OR, USA). Prior to anodization, the samples were degreased (acetone and ethanol) and electrochemically polished in a solution comprising HNO_3_ in ethanol (3:1 volumetric ratio).

Anodization was carried out in 20 wt.% H_2_SO_4_ in the voltage range from 10.0–22.5 V with step size of 2.5 V at 0 °C. After one minute at the first anodization step, the resulting poorly formed oxide was removed by chemical etching for 5 min in a vigorously stirred mixture of 6 wt.% H_3_PO_4_ and 1.8 wt.% H_2_CrO_4_ at 60 °C. After oxide removal, re-anodization was conducted under the same set of experimental conditions as the first step (one minute process).The anodizing process was carried out in a two-electrode system consisting of the FeAl_3_ intermetallic alloy anode and platinum cathode without any protection of inert gas.

The heat treatment process was carried out on previously anodized samples by annealing in a Nobotherm B170 tube furnace for 2 h at 900 °C in a protective argon atmosphere.The sample was heated up to 900 °C with heating rate 25 °C/min, then annealed for 2 h and cooled down with the furnace.

The phase analysis of the fabricated oxide film was carried out by the X-ray diffraction method using a Rigaku Ultima IV diffractometer with CoK_α_ radiation over the 2Θ range from 30°–90° with a step size of 0.01° and an acquisition rate of 1°/min. The crystallographic databases (PDF-2 2003 and PDF-4 + 2012) and PDXL software were used to identify the crystallographic phases.

X-ray photoelectron spectrometry(ESCA/XPS) was conducted on a PREVAC EA15 with a hemi-spherical analyzer to analyze the samples. XPS analyses of the samples were performed directly after the anodizing process. Either Al Kα (1486.6 eV) or Mg Kα (1253.7 eV) lamps were used for the analyses. The area of the analysis was 3 mm^2^, and the depth of the analysis was approximately 5 nm. Each time on the sample, measurements of the collective spectrum were performed with a sampling step of 0.25 eV and measurements of the detailed spectrum with a step of 0.05 eV.The spectra were calibrated for the C 1s excitation maximum at 285 eV. The deconvolution of the XPS spectra took into account the type of excitation, system, and excitation resolutions i.e., full width at half maximum, the number of components and constrains between them. In case of 2p doublet excitations, the intensity ratio of the 2p_3/2_ and 2p_1/2_ peaks was 2:1 and the excitations were separated by 13.5 eV for Fe and 0.41 eV for Al. The symmetric Voigt-type (70:30, Gauss: Lorentz) component profiles were applied in deconvolution procedure. The Shirley type of background was used for all spectra. The spectra were analyzed using Casa XPS 2.3.15.

To determine the energy gap of the produced anodic oxide coatings, spectrophotometric analysis was performed using a PERKIN ELMER LAMBDA 35 spectrophotometer with a reflexive attachment. The measurements were taken at room temperature, and spectra were recorded overthe wavelength range from 250 nm to 1100 nm. To determine the value of the band gap, the procedure by J. Tauca et al. [22] was used. On the basis of the shape of the absorption spectrum, the location of the absorption edge was determined, and then the band gap in the obtained anode oxide film was determined.

## 3. Results and Discussion

**Figure 1:**
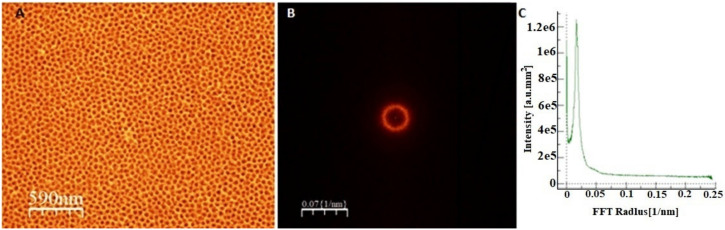
Top-down FE-SEM image (**A**), its fast Fourier transform (**B**), and radial average (**C**) of the oxide formed after two-step anodization of FeAl_3_ intermetallic alloy in 20 wt.% sulfuric acid at 0 °C and 20 V for 60 s.

To verify the homogeneity of the chemical composition of the cast material, which is the substrate material for the growth of the anodic coating in the volume of the ingot, microanalysis of the chemical composition in micro areas was performed using the EDS technique. The analysis was carried out, as in the case of crystallographic orientation tests, in three areas of the cast (i.e., on the top, in the middle, and on the bottom). This analysis showed the presence of two elements, iron (Fe) and aluminum (Al), in average atomic proportions of 77.5 at.% and 22.5 at.%, respectively. Based on the obtained results, it was found that the substrate material was chemically homogeneous in the whole volume of the FeAl_3_ intermetallic alloy ingot. The substrate material was electrochemically oxidized at different potentials in an aqueous solution of sulfuric (VI) acid for 60 s. The anodic coatings obtained in this way were subjected to chemical and phase composition analysis to fully identify the obtained porous coatings, an example of which is presented in Figure 1.

**Figure 2:**
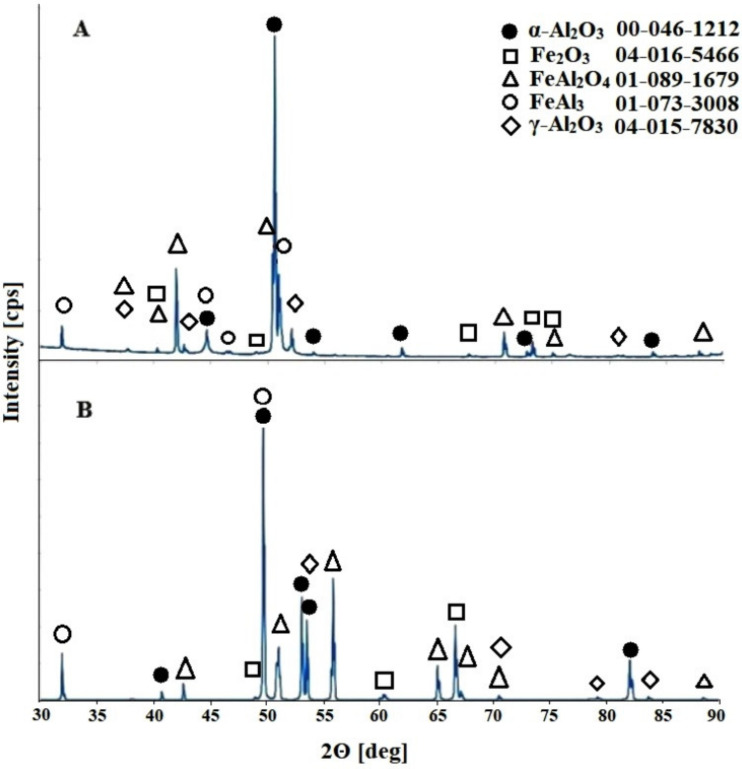
Diffraction patterns of the obtained anodic oxide on the FeAl_3_ substrate during anodization at a voltage of 10 V: (**A**) immediately after the process and (**B**) after the heat treatment.

An analysis of the diffraction pattern presented in Figure 2 indicates peaks originating from four crystal phases. One of the dominant phases found in the anodic oxide coating is Al_2_O_3_ and its γ-Al_2_O_3_ and α-Al_2_O_3_ allotropic forms. The next two phases that occur in the obtained coating are the FeAl_2_O_4_ spinel and Fe_2_O_3_ phase. It should be mentioned here that the unambiguous identification of the Fe_2_O_3_ phase was difficult because of the overlapping of its diffraction peaks with the peaks derived from the FeAl_2_O_4_ phase.

The diffraction peaks from the FeAl_3_ alloy, which is the substrate material, were also observed. The thickness of the coating varies depending on the voltage applied. These values are between 0.62 μm and 7.47 μm in extreme cases for 10 V and 22.5 V. This is precisely described in the previously published work [18]. Observed peaks coming from the substrate material may also provide the evidence of potential discontinuities in the resulting coating. It should also be noted that aluminum oxide occurs in crystalline form, even in the case of oxide coatings investigated directly after the anodizing process, for samples obtained over the entire range of applied anodizing potentials. This phenomenon is different than that observed during anodization of pure aluminum [21,22] or FeAl intermetallic alloys [16], which belongs to the same phase equilibrium system and where the amorphous structure of the resulting oxide coating is observed immediately after the anodization process. The subsequent heat treatment of the anodic oxide caused the crystallization of the phases present in the coating and enabled their identification, and the presence of FeAl_2_O_4_ iron–aluminum spinel was also found [18]. It should be noted that this phase, which is in the anode structure of the coating obtained on the FeAl alloy substrate that was subjected to annealing, was revealed at temperatures from 600 °C to 1000 °C. In addition, the anodic oxide obtained as a result of two-stage anodization of the FeAl alloy subjected to annealing in a protective atmosphere of argon did not contain peaks from Al_2_O_3_. The difference in the behavior of the oxide coatings obtained on the two intermetallic alloy substrates (FeAl and FeAl_3_) that belong to the same Fe–Al binary system is most likely related to their different chemical compositions Al content in the FeAl and FeAl_3_ alloys is 41.6 at.% and 77.5 at.%, respectively [15,18].

Moreover, the different mechanisms of the coating formation indicated by the crystal structures can be obtained directly after the anodization process of other metals, such as zirconium, where the crystal form of ZrO_2_ anodic oxide is observed [23]. In addition, the possibility of crystalline oxide formation was observed for technical grade aluminum anodized using the plasma electrolytic oxidation (PEO) technique [24]. As a result, two crystalline allotropic forms of aluminum oxide were obtained, namely, high-temperature α-Al_2_O_3_ [24], which has a high heat resistance, and hexagonal γ-Al_2_O_3_ [24,25], which has a relatively large specific surface and can be used as an adsorbent [26] or a catalyst carrier [27]. It is interesting that the described mechanism of the formation of the anodic alumina oxide layer as a result of the PEO process is similar to that observed during the anodization of FeAl_3_, as presented in this paper. A typical phenomenon for both processes is an almost immediate change in the color of the sample surface and the release of a large amount of gas that covers the entire surface of the sample. In addition, the value of the current density observed during anodization of the FeAl_3_ alloys (14 A/dm^2^) at the lowest applied voltage is more than three times higher than that used for the PEO of aluminum (4.4 A/dm^2^) [25]. Additionally, in both cases, the duration of the anodizing process is extremely short. During aluminum plasma anodization, the formation of single pores was observed after 20 s, and after 40 s, a porous oxide structure was obtained. However, during the anodization of the FeAl_3_ alloy, a homogeneous porous structure was obtained after a longer time of 60 s. The direct formation of a crystalline structure can also be caused by an increase in the local temperature in micro areas and weak heat transport during the process, which is related to the thickness of the oxide layer [25].

**Figure 3:**
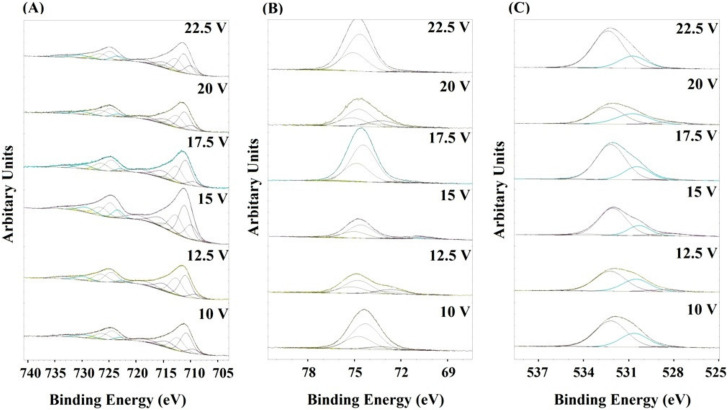
The Fe2p (**A**), Al2p (**B**), and O1s (**C**) core excitations for the FeAl_3_ surfaces obtained at different voltages.

X-ray photoelectron spectroscopy (XPS) was used to determine the chemical composition of the produced anodic oxide coatings. The measurements were carried out for all samples directly after the anodizing process (Figure S1) and after heat treatment of the fabricated oxides (Figure S2). The XPS analysis revealed Al, Fe, and O as the main surface components (Figure 3). The surface was also contaminated by an organic adsorbate from the ambient atmosphere. On the basis of the obtained spectra, one can conclude that depending on the conditions of the anodization process and precisely on the change in the applied voltage, a certain change in the atomic ratios of the given elements on the surface of the coating occurred. High-resolution spectra were obtained for all coatings subjected to the heat treatment in the same way as for the spectra obtained for the coatings immediately after the anodization process. However, by analyzing the spectral lines of iron, aluminum, and oxygen, it was found that the main surface component of the obtained anodic coating wasAl_2_O_3_. Nevertheless, the presence of iron species in the surface layer was also observed and assigned to Fe_2_O_3_, Fe_3_O_4_, or FeAl_2_O_4_ phases.

In the cases of Fe2p (Figure 3A), FeO (709.5 eV), Fe_2_O_3_/ Fe_3_O_4_ (710.6 eV), FeOH (713.8 eV), and satellites (714–720 eV) are observed [28,29] the evidence clearly demonstrates that the iron component is anodized during anodization. In Al2p (Figure 3B), two types of Al oxide, Al_2_O_3_ (74.4 eV) and AlOH (77.3 eV), are detected and FeAl_2_O_4_ (73.2 eV). The ratio of Al_2_O_3_ to AlOH clearly decreases as the anodizing voltage increases. In O1s (Figure 3C), peaks of FeOx (528.5 eV), Al_2_O_3_(531.8 eV), MOH (532.2 eV) are detected, confirming again the formation of a mixed oxide. The obtained XPS results are in good agreement with the XRD phase analysis where the presence of Al_2_O_3_, Fe_2_O_3_ and, FeAl_2_O_4_ is observed. An increase in the ratio of iron content to aluminum content (Fe% at/Al% at.) and the Fe concentration in the anode coating was observed together with an increase in the potential to 15 V, where both parameters reached the maximum value. A further increase in the anodizing potential caused decrease in both parameters again.

**Figure 4:**
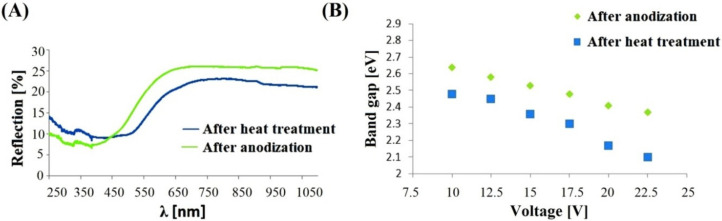
UV-Vis spectra after heat treatment and after anodization in 20 V (**A**); the band gap of the anodic oxide coatings produced on the FeAl_3_ alloy as a function of the applied voltage (**B**).

Visible and ultraviolet radiation absorption tests were performed on the anodic nanoporous oxides synthesized on the FeAl_3_ intermetallic alloy in the voltage range from 10 to 22.5 V. The spectra were obtained both for the anodic coatings obtained immediately after the process and for samples heat treated at 900 °C in an atmosphere of argon for two hours. Because of the large number of recorded spectra and the fact that they all showed similar relationships, only selected spectra are presented in this work. We measured the absorption wavelength of the anodic oxide using UV-VIS-IR spectroscopy, which indirectly indicates the tendency of the change in the energy band gap. The obtained UV-vis spectra were also used to determine the width of the band gap of the analyzed semiconductor materials. The absorption spectrum for visible and ultraviolet radiation presented in Figure 4A shows the original data of the spectrum in both cases, directly after the anodization process and after heat treatment. The changes in the band gap values for the fabricated coatings determined directly after anodizing and after heat treatment (Figure 4B) show a linear decrease with increasing voltage applied during the anodizing process.

The minimum band gap of 2.37 eV was obtained for the nanoporous oxide anodized at 22.5 V, while the maximum value of 2.64 eV was achieved on the anodic oxide produced at a potential of 10 V. A similar trend for the band gap was also observed for the anodic coating subjected to the heat treatment. In this case, a decrease in the value of the band gap was also observed, reaching a minimum of 2.10 eV at the highest anodizing voltage of 22.5 V and approaching a maximum at 2.48 eV for the coating produced at 10 V. It should be noted that the band gap for the anodic layer obtained at 17.5 V is the same as for the value obtained for the layer manufactured at 10 V and subsequently annealed. The value for both test variants, i.e., in the coating material obtained immediately after the anodizing process and after the heat treatment, is 2.48 eV.

In addition, the band gap value as a function of the voltage used during the formation of the oxide coating is similar for the oxide coating directly after anodization, after anodization and annealing, as well as for oxides produced on the FeAl substrate [16]. It should also be noted that the value of the band gap for the anodic oxide coating formed on the surface of the FeAl intermetallic alloy at a potential of 7.5 V (Eg = 2.43 eV) differs only by 0.2 eV compared to the band gap of the anodic oxide coating produced on the FeAl_3_ alloy at a potential of 10 V and heat treated (Eg = 2.45 eV). It should be noted that the chemical composition of the substrate materials is significantly different because the concentration of aluminum is 41.6 at.% and 77.5 at.% for FeAl [18] and FeAl_3_, respectively. In addition, it should be noted that in both cases, the anodic coatings were subjected to the annealing process, after which a slightly different phase composition was obtained. Namely, the anodic coating formed on the FeAl intermetallic alloy showed the presence of a single spinel phase (FeAl_2_O_4_) by XRD. The anodic oxide formed on the FeAl_3_ intermetallic alloy consisted of three oxide phases, namely, Fe_2_O_3_, FeAl_2_O_4_, and Al_2_O_3_.

Upon comparing the results obtained for the anodized FeAl intermetallic alloy and FeAl_3_ presented in this paper for anodic layers produced under similar process conditions, it can be stated that the anodic oxide obtained at the intermetallic alloy with higher contents of iron (crystal structure after heating) had lower band gap values than that obtained for the anodic oxide (crystal structure) produced on the alloy with a significantly higher aluminum content. These may be the effect of the elevated content of aluminum in the alloy, which leads to a mixture of oxides in the anode coating, a different proportion of individual oxides, and a band gap that is a function of the individual components of the oxide structure. The value of the band gap of individual phases identified in the coating is 4.3 eV for anodic Al_2_O_3_ produced in sulfuric acid [30], 2 eV for Fe_2_O_3_ [31], and a value from 3.51 eV to 2.09 eV for FeAl_2_O_4_ [15].

## 4. Conclusions

The research herein led to the effective production of a nanoporous coating using the anodization process for the first time on a construction material, which is the FeAl_3_ intermetallic alloy. It was shown that obtaining a crystal structure of nanoporous anodic oxide is possible immediately after processing, which is a novelty in the field of anodizing structural materials. The ability to control the band gap value of the produced anodic oxide coating was also obtained at the initial design stage of the material using a narrow technological window of the anodization process, and the value of the band gap makes this a feasible material for the titer of materials used for photocatalytic water splitting.

1. The phase composition of the produced anodic nanoporous coating is different from that obtained on aluminum or FeAl because it has a crystalline structure obtained directly after the anodization process.
2. By an appropriate selection of parameters of the anodization process, it is possible to control the band gap of the obtained coatings.
3. The value of the band gap of the produced coatings shows a linear decrease with increasing voltage applied during the anodizing process.
4. The values of the energy gap for the anodized coating subjected to annealing reach a minimum at 2.10 eV at an anodizing potential of 22.5 V and a maximum at 2.48 eV for a coating produced at a potential of 10 V.

## Supplementary Materials

The following are available online at https://www.mdpi.com/1996-1944/13/16/3471/s1, Figure S1: Collectiv spectra XPS for samples obtained directly after anodizing 10 V (bottom), 12.5 V, 15 V, 17.5 V, 20 V and 22.5 V (top), Figure S2: Collectiv spectra XPS for samples obtained heat treated 10V (bottom), 12.5 V, 15 V, 17.5 V, 20 V and 22.5 V (top).

## References

[1] Yasinskiy A.S., Padamata S.K., Polyakov P.V., Shabanov A.V. (2020). An update on inert anodes for aluminium electrolysis. Non Ferr. Met..

[2] Yang Y., Albu S.P., Kim D., Schmuki P. (2011). Enabling the anodic growth of highly ordered V_2_O_5_nanoporous/nanotubular structures. Angew. Chem. Int. Ed..

[3] Salerno M., Stępniowski W., Cieślak G., Norek M., Michalska-Domańska M., Karczewski K., Chilimoniuk P., Polkowski W., Jóźwik P., Bojar Z. (2016). Advanced image analysis of the surface pattern emerging in Ni_3_Al intermetallic alloys on anodization. Front. Mater..

[4] Stepniowski W.J., Cieslak G., Norek M., Karczewski K., Michalska-Domanska M., Zasada D., Polkowski W., Józwik P., Bojar Z. (2013). Nanoporous alumina formed by self-organized two-step anodization of Ni_3_Al intermetallic alloy in citric acid. Appl. Surf. Sci..

[5] Tsuchiya H., Berger S., Macak J.M., Ghicov A., Schmuki P. (2007). Self-organized porous and tubular oxide layers on TiAl alloys. Electrochem. Commun..

[6] Wu L.-K., Xia J.-J., Cao H.-Z., Liu W.-J., Hou G.-Y., Tang Y.-P., Zheng G.-Q. (2018). Improving the high-temperature oxidation resistance of TiAl alloy by anodizing in Methanol/NaF solution. Oxid. Met..

[7] Lohrengel M.M. (1993). Thin anodic oxide layerson aluminium and other valve metals: High field regime. Materials Science and Engineering: R: Reports.

[8] Alkire R.C., Kolb D.M. (2008). Advances in Electrochemical Science and Engineering.

[9] Revie R.W. (2011). Uhlig’s Corrosion Handbook.

[10] Sulka G.D., Zaraska L., Stępniowski W.J. (2011). Anodic Porous Alumina as a Template for Nanofabrication. Encyclopedia of Nanoscience and Nanotechnology.

[11] Sulka G.D., Stępniowski W.J. (2009). Structural features of self-organized nanopore arrays formed by anodization of aluminum in oxalic acid at relatively high temperatures. Electrochim. Acta.

[12] Zaraska L., Sulka G.D., Jaskuła M. (2009). Properties of nanostructures obtained by anodizations of aluminum in phosphoric acid at moderate potentials. J. Phys. Conf. Ser..

[13] Chernyakova K., Tzaneva B., Vrublevsky I., Videkov V. (2020). Effect of aluminum anode temperature on growth rate and structure of nanoporous anodic alumina. J. Electrochem. Soc..

[14] Stępniowski W., Choi J., Yoo H., Michalska-Domańska M., Chilimoniuk P., Czujko T. (2016). Quantitative fast Fourier transform based arrangement analysis of porous anodic oxide formed by self-organized anodization of FeAl intermetallic alloy. Mater. Lett..

[15] Stępniowski W., Choi J., Yoo H., Oh K., Michalska-Domańska M., Chilimoniuk P., Czujko T., Łyszkowski R., Jóźwiak S., Bojar Z. (2016). Anodization of FeAl intermetallic alloys for bandgap tunable nanoporousmixed aluminum-iron oxide. J. Electroanal. Chem..

[16] Chilimoniuk P., Michalska-Domańska M., Stępniowski W., Czujko T. (2018). Formation of nanoporous oxide byself-organized anodizing of FeAl intermetallic alloy in oxalic acid solution containing glycol. Mat. Lett..

[17] Sulka G.D., Parkoła K.G. (2007). Temeprature influence on well-ordered nanopore structures grown by anodization of aluminium in sulphuric acid. Electrochim. Acta.

[18] Chilimoniuk P., Michalska-Domańska M., Czujko T. (2019). Formation of nanoporous mixed aluminum-iron oxides by self-organized anodizing of FeAl_3_ intermetallic alloy. Materials.

[19] Stępniowski W.J., Norek M., Michalska-Domańska M., Bojar Z. (2013). Ultra-small nanopores obtained by self-organized anodization of aluminum in oxalic acid at low voltages. Mater. Lett..

[20] Masuda T., Asoh H., Haraguchi S., Ono S. (2015). Fabrication and characterization of single phase α-alumina membranes with tunable pore diameters. Procedia Manuf..

[21] Losic D., Santos A. (2015). Nanoporous Alumina Fabrication, Structure, Properties and Applications.

[22] Tauc J., Grigorovici R., Vancu A. (1966). Optical properties and electronic structure of amorphous germanium. Phys. Status Solidi..

[23] Lockmana Z., Abidina N.R.Z., Ismaila S., Cheonga K.Y., Hassanb Z. (2012). Effects of applied voltage on the properties of anodic zirconia thinfilm on (100) silicon. Thin Solid Film..

[24] Wang D., Liu X., Wu Y., Han H., Yang Z., Su Y., Zhang X., Wu G., Shen D. (2019). Evolution process of the plasma electrolytic oxidation (PEO) coating formed on aluminum in an alkaline sodium hexametaphosphate ((NaPO_3_)_6_) electrolyte. J. Alloy. Compd..

[25] Wang K., Koo B.-H., Lee C.-G., Kim Y.-J., Lee S.-H., Byon E. (2009). Effects of electrolytes variation on formation of oxide layers of 6061 Al alloys by plasma electrolytic oxidation. Trans. Nonferrous Met. Soc. China.

[26] Bielański A. (2002). Podstawy Chemii Nieorganicznej.

[27] Schauermann S., Hoffmann J., Johanek V., Hartmann J., Libuda J., Freund H.-J. (2002). The molecular origins of selectivity in methanol decomposition on Pd nanoparticles. Catal. Lett..

[28] Aronniemi M., Lahtinen J., Hautojärvi P. (2004). Characterization of iron oxide thin films. Surf. Interface Anal..

[29] Mills P., Sullivan J.L. (1983). A study of the core level electrons in iron and its three oxides by means of X-ray photoelectron spectroscopy. J. Phys. D Appl. Phys..

[30] Ramanareddy P., Ajith K.M., Udayashankar N.K. (2018). Optical and mechanical studies on free standing amorphous anodic porous alumina formed in oxalic and sulphuric acid. Appl. Phys. A.

[31] Mallick P., Dash B.N. (2013). X-ray diffraction and UV-visible characterizations of α-Fe_2_O_3_ nanoparticles annealed at different temperature. Nanosci. Nanotechnol..

