# Supplementary material for: Nanoporous Anodic Aluminum-Iron Oxide with a Tunable Band Gap Formed on the FeAl3 Intermetallic Phase
Source: Materials (Basel). 2020 Aug 6;13(16):3471. doi: 10.3390/ma13163471 (PMC7475830; doi:10.3390/ma13163471)
Supplement: Supplementary file 1 [file materials-13-03471-s001.pdf]

materials

Article

Nanoporous Anodic Aluminum-Iron Oxide with a Tunable Band Gap Formed on the FeAl3 Intermetallic Phase

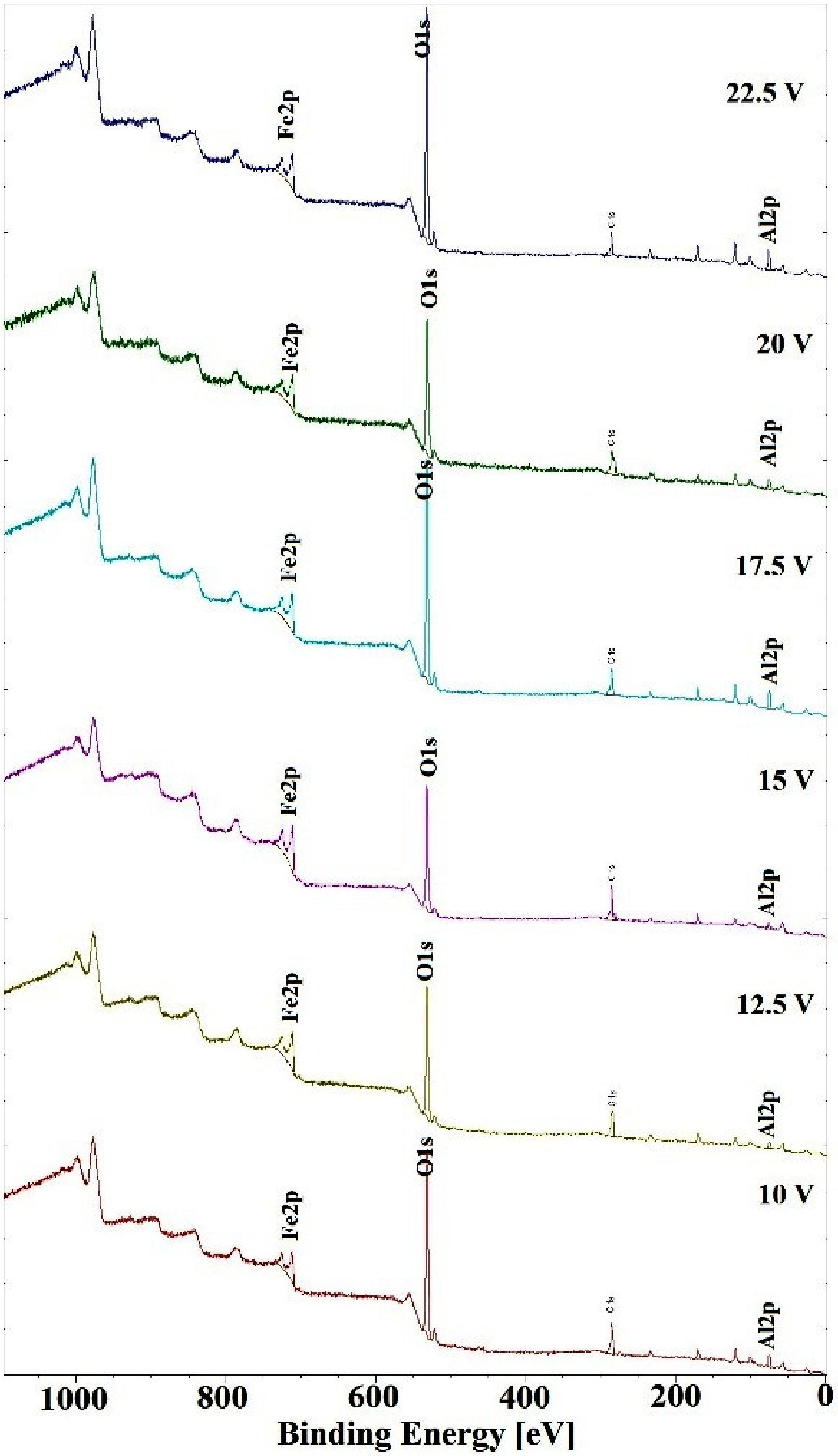

Figure S1. Collectiv spectra XPS for samples obtained directly after anodizing 10 V (bottom), 12.5 V, 15 V, 17.5 V, 20 V and 22.5 V (top).

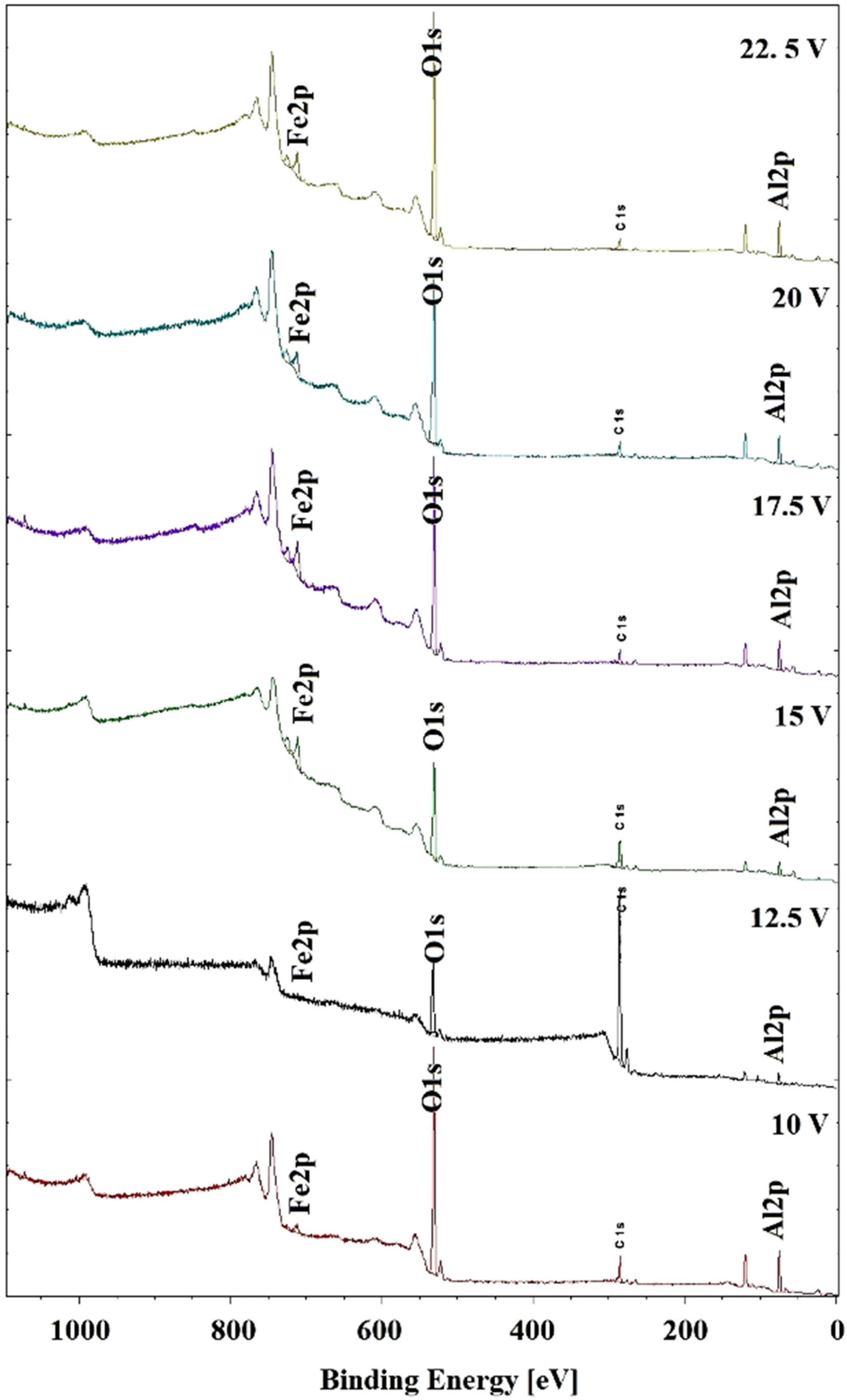

Figure S2. Collectiv spectra XPS for samples obtained heat treated 10 V (bottom), 12.5 V, 15 V, 17.5 V, 20 V and 22.5 V (top).

© 2020 by the authors. Submitted for possible open access publication under the terms and conditions of the Creative Commons Attribution (CC BY) license (http://creativecommons.org/licenses/by/4.0/).
